# Influenza: an emerging disease.

**DOI:** 10.3201/eid0403.980325

**Published:** 1998

**Authors:** R G Webster

**Affiliations:** St. Jude Children's Research Hospital, Memphis, TN 38105-2794, USA. robert.webster@stjude.org

## Abstract

Because all known influenza A subtypes exist in the aquatic bird reservoir, influenza is not an eradicable disease; prevention and control are the only realistic goals. If people, pigs, and aquatic birds are the principal variables associated with interspecies transfer of influenza virus and the emergence of new human pandemic strains, influenza surveillance in these species is indicated. Live-bird markets housing a wide variety of avian species together (chickens, ducks, geese, pigeon, turkeys, pheasants, guinea fowl), occasionally with pigs, for sale directly to the public provide outstanding conditions for genetic mixing and spreading of influenza viruses; therefore, these birds should be monitored for influenza viruses. Moreover, if pigs are the mixing vessel for influenza viruses, surveillance in this population may also provide an early warning system for humans.


### Influenza: An Emerging Disease

**Robert G. Webster**
St. Jude Children's Research Hospital, Memphis, Tennessee, USA

Because all known influenza A subtypes exist in the aquatic bird reservoir, influenza is not an eradicable disease; prevention and control are the only realistic goals. If people, pigs, and aquatic birds are the principal variables associated with interspecies transfer of influenza virus and the emergence of new human pandemic strains, influenza surveillance in these species is indicated. Live-bird markets housing a wide variety of avian species together (chickens, ducks, geese, pigeon, turkeys, pheasants, guinea fowl), occasionally with pigs, for sale directly to the public provide outstanding conditions for genetic mixing and spreading of influenza viruses; therefore, these birds should be monitored for influenza viruses. Moreover, if pigs are the mixing vessel for influenza viruses, surveillance in this population may also provide an early warning system for humans.

The influenza virus continues to evolve, and new antigenic variants (drift strains) emerge constantly, giving rise to yearly epidemics. In addition, strains to which most humans have no immunity appear suddenly, and the resulting pandemics vary from serious to catastrophic.

Influenza viruses are unique among respiratory tract viruses in that they undergo considerable antigenic variation. Both surface antigens of the influenza A viruses undergo two types of variation: drift and shift (1). Antigenic drift involves minor changes in the hemagglutinin (HA) and neuraminidase (NA), whereas antigenic shift involves major changes in these molecules resulting from replacement of the gene segment.

### The Reservoirs of Influenza A Viruses

Aquatic birds are the reservoirs of all 15 subtypes of influenza A viruses. In wild ducks, influenza viruses replicate preferentially in the cells lining the intestinal tract, cause no disease signs, and are excreted in high concentrations in the feces (up to $10^{8.7}$ 50% egg infectious doses/g) (2). Avian influenza viruses have been isolated from freshly deposited fecal material and from unconcentrated lake water, which indicates that waterfowl have a very efficient way to transmit viruses, i.e., by fecal material in the water supply. Since a large number of susceptible young ducks are hatched each year throughout the world, many birds are infected by virus shed into water. This would explain the high incidence of virus infection in Canadian ducks, particularly juveniles, when up to 30% can shed virus before fall migration. Transmission by feces also provides a way for wild ducks as they migrate through an area to spread their viruses to other domestic and feral birds (3).

The avirulent nature of avian influenza infection in ducks and wading birds may result from virus adaptation to this host over many centuries, which created a reservoir that ensures perpetuation of the virus; therefore, ducks and wading birds may be occupying an important position in the natural history of influenza viruses. Influenza viruses of avian origin have been implicated in outbreaks of influenza in mammals, such as seals (4), whales (5), and pigs (6), as well as in domestic poultry (7).

### Evolutionary Pathways for Influenza Viruses

Studies on the ecology of influenza viruses have led to the hypothesis that all mammalian influenza viruses derive from the avian influenza reservoir. Support for this theory comes from phylogenetic analyses of nucleic acid sequences of influenza A viruses from a variety of hosts, geographic regions, and virus subtypes. Analyses of the nucleoprotein (NP) gene show that avian influenza viruses have evolved into five host-

Address for correspondence: Robert G. Webster, St. Jude Children's Research Hospital, 332 North Lauderdale St., Memphis TN 38105-2794, USA; fax: 901-523-2622; e-mail: robert.webster@stjude.org.

specific lineages: ancient equine, which has not been isolated in over 15 years; recent equine; gull; swine; and human. The human and classic swine viruses have a genetic "sister group" relationship, which shows that they evolved from a common origin. The ancestor of the human and classic swine virus appears to have been an intact avian virus that, like the influenza virus currently circulating in pigs in Europe, derived all its genes from avian sources (8,9).

Studies on the NP and other gene lineages in avian species show separate sublineages of influenza in Eurasia and the Americas, indicating that migratory birds moving between these continents (latitudinal migration) have little or no role in the transmission of influenza, while birds that migrate longitudinally appear to play a key role in the continuing process of virus evolution.

Phylogenetic analyses of amino acid changes show that avian influenza viruses, unlike mammalian strains, have low evolutionary rates (8). In fact, influenza viruses in aquatic birds appear to be in evolutionary stasis, with no evidence of net evolution over the past 60 years. Nucleotide changes have continued at a similar rate in avian and mammalian influenza viruses; however, these changes no longer result in amino acid changes in the avian viruses, whereas all eight mammalian influenza gene segments continue to accumulate changes in amino acids. The high level of genetic conservation suggests that avian viruses are approaching or have reached optimum, wherein nucleotide changes provide no selective advantage. It also means that the source of genes for pandemic influenza viruses exists phenotypically unchanged in the aquatic bird reservoir. The most important implication of phylogenetic studies is that the ancestral viruses that caused the Spanish flu in 1918, as well as the viruses that provided gene segments for the Asian/1957 and Hong Kong/1968 pandemics, are still circulating in wild birds, with few or no mutational changes.

### Emergence and Reemergence of "New" Influenza A Virus in Humans

Over the past two and a half centuries, 10 to 20 human influenza pandemics have swept the globe; the most devastating, the so-called Spanish flu of 1918 to 1919, caused more than 20 million deaths and affected more than 200 million people. Both pandemics probably originated from aquatic birds.

Since the first human influenza virus was isolated in 1933, new subtypes of human type A influenza viruses have occurred: H2N2 (Asian influenza) replaced H1N1 in 1957, Hong Kong (H3N2) virus appeared in 1968, and H1N1 virus reappeared in 1977. Each of these new subtypes first appeared in China, and anecdotal records suggest that previous epidemics also had their origin in China. Serologic and virologic evidence suggests that since 1889 there have been six instances of the introduction of a virus bearing an HA subtype that had been absent from the human population for some time. Three human subtypes of HA have appeared cyclically—H2 viruses in 1889, H3 in 1900, H1 in 1918, H2 again in 1957, H3 again in 1968, and H1 again in 1977. Phylogenetic evidence indicates that a totally new H1N1 virus of avian origin (not a reassortant) could have appeared in humans or swine before the 1918 influenza and replaced the previous human virus strains. Whether the virus was first introduced into humans and then transmitted to pigs, or vice versa, remains unknown. The reappearance of the H1N1 Russian 1977 influenza virus remains a mystery.

### How Are Influenza Viruses Spread?

Avian influenza viruses in wild aquatic birds are spread by fecal-oral transmission through the water supply (10); initial transmission of avian influenza viruses to mammals, including pigs and horses, probably also occurs by fecal contamination of water. Scholtissek has postulated that the use of fecal material from ducks for fish farming in Asia may contribute to transmission of avian influenza viruses to pigs (11). Another direct method of transfer is by feeding pigs untreated garbage or the carcasses of dead birds. Raising pigs under chicken houses and feeding them dead avian carcasses has been observed on rare occasions in the United States; H5N2 influenza virus was isolated from pigs living under chicken houses in Pennsylvania during the outbreak in 1982. Both pigs and poultry are commonly raised on the same commercial farms. From the perspective of the control of interspecies transmission of influenza, this is undesirable, for it may facilitate interspecies transmission of influenza viruses. After transmission to pigs, horses, or humans, the method of spread of influenza is mainly respiratory.

### Emergence of H5N2 Influenza Viruses in North America

In 1983 an H5N2 influenza virus infected chickens and turkeys in Pennsylvania and became highly pathogenic for poultry. Virologic and serologic studies provided no evidence of transmission to humans (12). The virus was eventually eradicated by quarantine and extermination of more than 17 million birds at a direct cost of more than US$60 million and an indirect cost to the industry of more than US$250 million.

More recently, a highly pathogenic H5N2 influenza virus emerged in domestic chickens in Mexico (7). In October 1993, egg production decreased and deaths increased among Mexican chickens in association with serologic evidence of an H5N2 influenza virus. H5N2 virus was isolated in May 1994. By the end of 1994, the virus had mutated to contain a highly cleavable HA, but remained only mildly pathogenic in chickens. Within months, however, it had become lethal in poultry. Phylogenetic analysis of the HA of H5 avian influenza viruses, including the Mexican isolates, indicated that the epidemic virus had originated from the introduction of a single virus of the North American lineage into Mexican chickens (Figure 1). This virus was eradicated from chickens by quarantine and use of inactivated vaccine.

### Live Bird Markets and the Epidemiology of Influenza

The chicken/Pennsylvania (H5N2) influenza outbreak in 1983 to 1984 demonstrated that live bird markets play an important part in the spread of influenza viruses in avian species. In 1992, Senne et al. (13) described live bird markets as the "missing link in the epidemiology of avian influenza," for H5N2 viruses had been isolated from live birds until 1986. These H5N2 viruses caused subclinical infection in chickens in the markets, as did H5N1 viruses in live bird markets in Hong Kong in 1997 (Figure 2). Moreover, ducks in the markets in the United States were infected with many different subtypes of influenza A viruses, including H2N2 viruses related antigenically to the Asian/57 (H2N2) viruses that have disappeared from humans.

The live bird markets in the United States continue to harbor many influenza viruses. The ancestor of the H5N2 influenza virus that caused the epidemic in Mexico in 1993 to 1995 was isolated from market birds, and H7NX subtypes are still found in live bird markets. These viruses are potentially pathogenic for chickens and are of

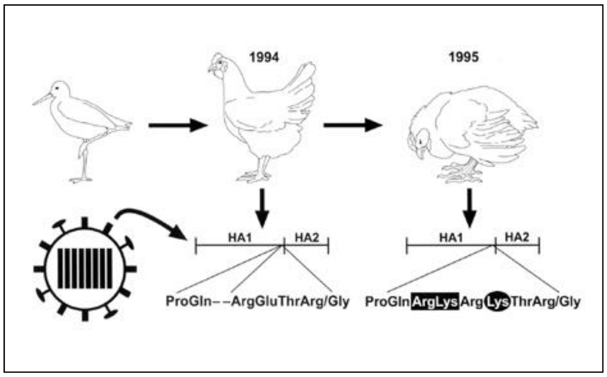

Figure 1. Molecular changes associated with emergence of a highly pathogenic H5N2 influenza virus in chickens in Mexico. In 1994, a nonpathogenic H5N2 influenza virus in Mexican chickens was related to an H5N2 virus isolated from shorebirds (ruddy turnstones) in Delaware Bay, United States, in 1991. The 1994 H5N2 isolates from chickens replicated mainly in the respiratory tract, spread rapidly among chickens, and were not highly pathogenic. Over the next year the virus became highly pathogenic, and the hemagglutinin acquired an insert of two basic amino acids (Arg-Lys), possibly by classic recombination and a mutation of Glu to Lys at position – 3 from the cleavage site of HA1/HA2.

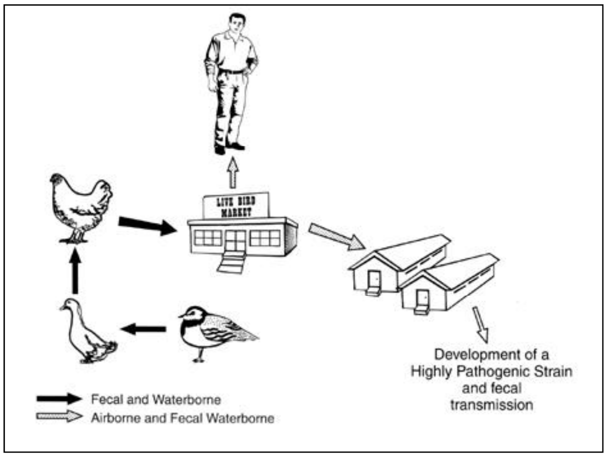

Figure 2. The emergence of H5N1 influenza in Hong Kong. It is postulated that a nonpathogenic H5N1 influenza spread from migrating shorebirds to ducks by fecal contamination of water. The virus was transmitted to chickens and became established in live bird markets in Hong Kong. During transmission between different species, the virus became highly pathogenic for chickens and occasionally was transmitted to humans from chickens in the markets. Despite high pathogenicity for chickens (and humans), H5N1 were nonpathogenic for ducks and geese.

great concern to chicken farmers in the northeastern United States.

The depopulation of live bird markets and farms in the New Territories of Hong Kong (December 29, 1997) stopped the spread of H5N1 influenza viruses. An important lesson can be learned from this action in Hong Kong. Live bird markets are potential breeding grounds for both avian and mammalian influenza viruses. Serologic monitoring of the chickens in Hong Kong markets for H5N1 influenza virus was an important first step in stopping the spread of the viruses. An even more important step would be to reduce the opportunity for interspecies transmission by marketing chickens separately from other avian species.

### The Index Case of H5N1 in Humans in Hong Kong

On May 21, 1997, a 3-year-old boy from Hong Kong died in an intensive care unit in Hong Kong on the fifth day of his hospitalization, with a final diagnosis of Reye syndrome, acute influenza pneumonia, and respiratory distress syndrome (14). He had no indications of other underlying disease, including immunodeficiency or cardiopulmonary disease. From a tracheal aspirate, we isolated an influenza virus in MDCK cells but were unable to grow any pathogenic bacteria from respiratory specimens. In hemagglutination inhibition assays, the virus did not react with ferret antisera to recent isolates of human and swine subtypes.

Hemagglutination inhibition assays using antisera to 14 H subtypes showed that the isolate was an H5 influenza A virus. Neuraminidase inhibition tests, using antisera to nine N subtypes, indicated that the neuraminidase was of the N1 subtype. Nucleotide sequence analyses of parts of the HA and NA genes of the virus allowed a phylogenetic comparison with other influenza viruses. Our analyses confirmed that the virus was of the H5N1 subtypes. Each of the eight RNA segments wase of avian origin, and the virus was highly pathogenic for chickens. The contribution of the influenza A H5N1 virus infection to the child's disease, eventually leading to death, was complicated by the child's treatment with aspirin. The virus identification is important because it is the first documented isolation of an influenza A virus of this subtype from humans (15).

### Characterization of the Human and Chicken H5N1 Viruses from Hong Kong

Avian influenza outbreaks occurred in Hong Kong from late March to early May of 1997. Three chicken farms were separately affected; the death rate for the total of 6,800 chickens exceeded 70%. A comparison between the nucleotide sequences of the H5 genes from both the human virus A/Hong Kong/156/97 (H5N1) (HK97) and a representative of the chicken viruses from the March outbreak, A/chicken/Hong Kong/258/97 (CkHK97), showed a high degree of homology in their respective H5 HA1 sequences. Only three amino-acid differences were observed in the HA1 of the HA, confirming the close phylogenetic relationship between these viruses, belonging to the Eurasian lineage of the subtype H5 viruses.

Sequence analyses of the HA of multiple human and chicken H5N1 isolates show that they form two subgroups with close linkage between chicken and human isolates. An analysis of the amino acids expected to be involved in the assembly of the receptor binding site showed no differences could be observed between the human isolate and avian H5 viruses. Therefore, the H5 HA of HK97 had probably not acquired mutations that favor binding to sialic acids with 2,6 linkage to the galactoside over the 2,3 linked sialic acid receptor preferred by human and avian viruses respectively. However, the loss of a potential N-linked glycosylation site at amino acid 156 Asn, close to the receptor binding site, could affect binding to the cellular receptor.

The amino acid sequence motif at the cleavage site of the HA molecule has been associated with high virulence of avian influenza viruses. Experimental infection of chickens with HK97 showed that even after passaging in mammalian cells (once in the child and twice in MDCK cells), the virus remained highly pathogenic for chickens: all eight chickens inoculated intratracheally with MDCK-grown HK97 died within 3 days after infection. A comparison of the reactivity of a panel of 17 monoclonal antibodies (MAb) directed against A/chicken/Pennsylvania/83 (H5N2) with HK97 and CkHK97 in a hemagglutination inhibition assay showed similar antigenic reactivities with all but one MAb, indicating antigenic cross-reactivity between these viruses and the usefulness of these antibodies for diagnosis.

The fetuin-cleaving activity of the NA proved to be inhibited by anti-NA antiserum. Reverse transcriptase polymerase chain reaction using primer sets that amplified the 5' end of the NA gene segments showed that this gene was of the N1 genotype. Nucleotide sequence analysis and comparison to published NA sequences confirmed this finding genetically. The NA sequences unequivocally showed a close molecular relationship between HK97 and CkHK97, as a unique 57-nucleotide deletion was observed in the stalk region of the N1 gene of both viruses. Each of the eight gene segments showed close genetic homology between the HK97 and Ck/HK97 viruses, the lowest being 98.2% for the nucleoprotein; the remaining genes varied from 98.8% to 100% homology (16).

### Can the Emergence of Pandemic Strains Be Prevented?

Because all known influenza A virus subtypes are found in aquatic wild birds in nature, agricultural authorities have recommended avoiding direct or indirect contact between domestic poultry and wild birds. A classic mistake made by chicken and turkey farmers is to raise a few domestic ducks on a pond near poultry barns; these birds attract wild ducks. The highly pathogenic outbreaks of H5N2 avian influenza in chickens and turkeys in Pennsylvania and surrounding states in 1983 to 1984 (12) and the H5N2 in Mexico in 1993 (7) could probably have been prevented if domestic poultry had been raised in ecologically controlled houses with a high standard of security and limited access.

If we assume that people, pigs, and aquatic birds are the principal variables associated with the emergence of new human pandemic stains, human pandemics of influenza may be prevented. The principles applied to preventing outbreaks of influenza in domestic animals should apply equally here. Pandemic strains of human influenza emerge only rarely; however, interspecies transmission of influenza viruses may not be so rare, for up to 10% of persons with occupational exposure to pigs develop antibodies to swine influenza virus (17). Most transfers of influenza viruses from pigs to humans are dead-end transfers (they do not spread efficiently from human to human). As indicated above, we do not know the frequency of virus transfer between the suspect species in southern China. If there is an epicenter for pandemic influenza and a detectable frequency of transfer between people, pigs, and ducks and if we understand the ecologic and agricultural features involved in the transfer, pandemics may be preventable. If pigs are the major mixing vessel for influenza viruses, changes in the agricultural practices that separate pigs from people and ducks could prevent future pandemics. Most importantly, we may influence the appearance of pandemics by changing the methods of live bird marketing by separating chickens from other species, especially from aquatic birds.

This work was supported by Public Health Service grants AI-29680 and AI-08831 from the National Institute of Allergy and Infectious Diseases, by Cancer Center Support (CORE) grant CA-21765, and by the American Lebanese Syrian Associated Charities.

Dr. Webster holds the Rose Marie Thomas Chair in the Department of Virology and Molecular Biology at St. Jude Children's Research Hospital, Memphis, Tennessee. In addition, he is director of the World Health Organization Collaborating Center for Ecology of Influenza Viruses in Lower Animals and Birds. He has devoted his life to the understanding of the emergence of pandemic influenza viruses, the structure and function of the viral proteins, and methods for developing new and improved antiviral drugs and vaccines.

### References

1. Murphy BR, Webster RG. Orthomyxoviruses. In: Fields BN, Knipe DM, Howley PM, Chanock RM, Melnick JL, Monath TP, Roizman R, Straus SE, editors. Fields virology. New York: Raven Press; 1996. p. 1397-445.
2. Webster RG, Yakhno MA, Hinshaw VS, Bean WJ, Murti KG. Intestinal influenza: replication and characterization of influenza viruses in ducks. Virology 1978;84:268-78.
3. Halvorson D, Karunakaran D, Senne D, Kelleher C, Bailey C, Abraham A, et al. Epizootiology of avian influenza-simultaneous monitoring of sentinel ducks and turkeys in Minnesota. Avian Dis 1983;27:77-85.
4. Geraci JR, St. Aubin DJ, Barker IK, Webster RG, Hinshaw VS, Bean WJ, et al. Science 1982;215:1129-31.
5. Hinshaw VS, Bean WJ, Geraci JR, Fiorelli P, Early G, Webster RG. Characterization of two influenza A viruses from a pilot whale. J Virol 1986;58:655-6.
6. Scholtissek C, Burger H, Bachmann PA, Hannoun C. Genetic relatedness of hemagglutinins of the H1 subtype of influenza A viruses isolated from swine and birds. Virology 1983;129:521-3.
7. Horimoto T, Rivera E, Pearson J, Senne D, Krauss S, Kawaoka Y, et al. Origin and molecular changes associated with emergence of a highly pathogenic H5N2 influenza virus in Mexico. Virology 1995;213:223-30.

8. Gorman OT, Bean WJ, Kawaoka Y, Webster RG. Evolution of the nucleoprotein gene of influenza A virus. J Virol 1990;64:1487-97.
9. Gammelin M, Altmuller A, Reinhardt U, Mandler J, Harley VR, Hudson PJ, et al. Phylogenetic analysis of nucleoproteins suggests that human influenza A viruses emerged from a 19th-century avian ancestor. Mol Biol Evol 1990;7:194-200.
10. Hinshaw VS, Webster RG. The natural history of influenza A viruses. In: Beare AS, editor. Basic and applied influenza research. Boca Raton (FL): CRC Press; 1982. p. 79-104.
11. Scholtissek C, Naylor E. Fish farming and influenza pandemics. Nature 1988;331:215.
12. Bean WJ, Kawaoka Y, Wood JM, Pearson JE, Webster RG. Characterization of virulent and avirulent A/Chicken/Pennsylvania/83 influenza A viruses: potential role of defective interfering RNAs in nature. J Virol 1985;54:151-60.
13. Senne DA, Pearson JE, Panigrahy B. Live poultry markets: a missing link in the epidemiology of avian influenza. In: Proceedings of the 3rd International Symposium on Avian Influenza; 1997 27-29 May; The Wisconsin Center, The University of Wisconsin-Madison. p. 50-8.
14. De Jong JC, Claas ECJ, Osterhaus ADME, Webster RG, Lim WL. A pandemic warning. Nature 1997;389:554.
15. Subbarao K, Klimov A, Katz J, Regnery H, Lim W, Hall H, et al. Characterization of an Avian influenza A (H5N1) virus isolated from a child with a fatal respiratory illness. Science 1998;279:393-6.
16. Claas ECJ, Osterhaus ADME, van Beek R, De Jong JC, Rimmelzwaan GF, Senne DA, et al. Human influenza A H5N1 virus related to a highly pathogenic avian influenza virus. Lancet 1998;351:472-7.
17. Schnurrenberger PR, Woods GT, Martin RJ. Serologic evidence of human infection with swine influenza virus. Am Rev Respir Dis 1970;102:356-61.
